# Metabolic Dysfunction‐Associated Steatotic Liver Disease After Hepatitis C Virus Cure

**DOI:** 10.1002/kjm2.70155

**Published:** 2025-12-10

**Authors:** Chung‐Feng Huang, Jee‐Fu Huang, Ming‐Lung Yu, Wan‐Long Chuang

**Affiliations:** ^1^ Hepatobiliary Division, Department of Internal Medicine Kaohsiung Medical University Hospital, Kaohsiung Medical University Kaohsiung Taiwan; ^2^ Graduate Institute of Clinical Medicine Kaohsiung Medical University Kaohsiung Taiwan; ^3^ Center for Metabolic Disorder and Obesity Kaohsiung Medical University Kaohsiung Taiwan; ^4^ Center of Excellence for Metabolic Associated Fatty Liver Disease National sun Yat‐Sen University Kaohsiung Taiwan

**Keywords:** cardiometabolic risk factors, hepatitis C virus, metabolic dysfunction‐associated steatotic liver disease, steatotic liver disease, sustained virological response

## Abstract

Hepatitis C virus (HCV) infection is an infectious disease carrying a high risk of metabolic disorders. Chronic HCV (CHC) patients can possess extrahepatic manifestations such as diabetes, steatotic liver disease (SLD), and other metabolic alterations. Hepatic steatosis, which is triggered by insulin resistance or the direct insult of a specific genotype, is commonly observed in CHC. The changes in SLD after HCV eradication by direct‐acting antivirals (DAAs) present a significant impact on patient outcomes. We reviewed the changes in cardiometabolic risk factors (CMRFs) and the evolution of CMRFs items in CHC patients with SLD after HCV cure. The reassessment of CMRFs after the successful eradication of HCV is an essential task for primary care providers. CHC patients could benefit from appropriate management of both infectious and metabolic disorders in terms of liver and nonliver related outcomes.

**Abbreviations**
BMI: body mass index
CHC: chronic hepatitis C
CMRF: cardiometabolic risk factors
CVD: cardiovascular disease
DAAs: direct acting antivirals
HbA1c: hemoglobin A1c
HCV: hepatitis C virus
IR: insulin resistance
LDL: low‐density lipoprotein
LDL‐C: low‐density lipoprotein cholesterol
MAFLD: metabolic dysfunction‐associated fatty liver disease
MASLD: metabolic dysfunction‐associated steatotic liver disease
NAFLD: nonalcoholic fatty liver disease
PNPLA3: patatin‐like phospholipase domain‐containing 3
SLD: steatotic liver disease
SVR: sustained virological response

## 1 Introduction

**FIGURE 1:**
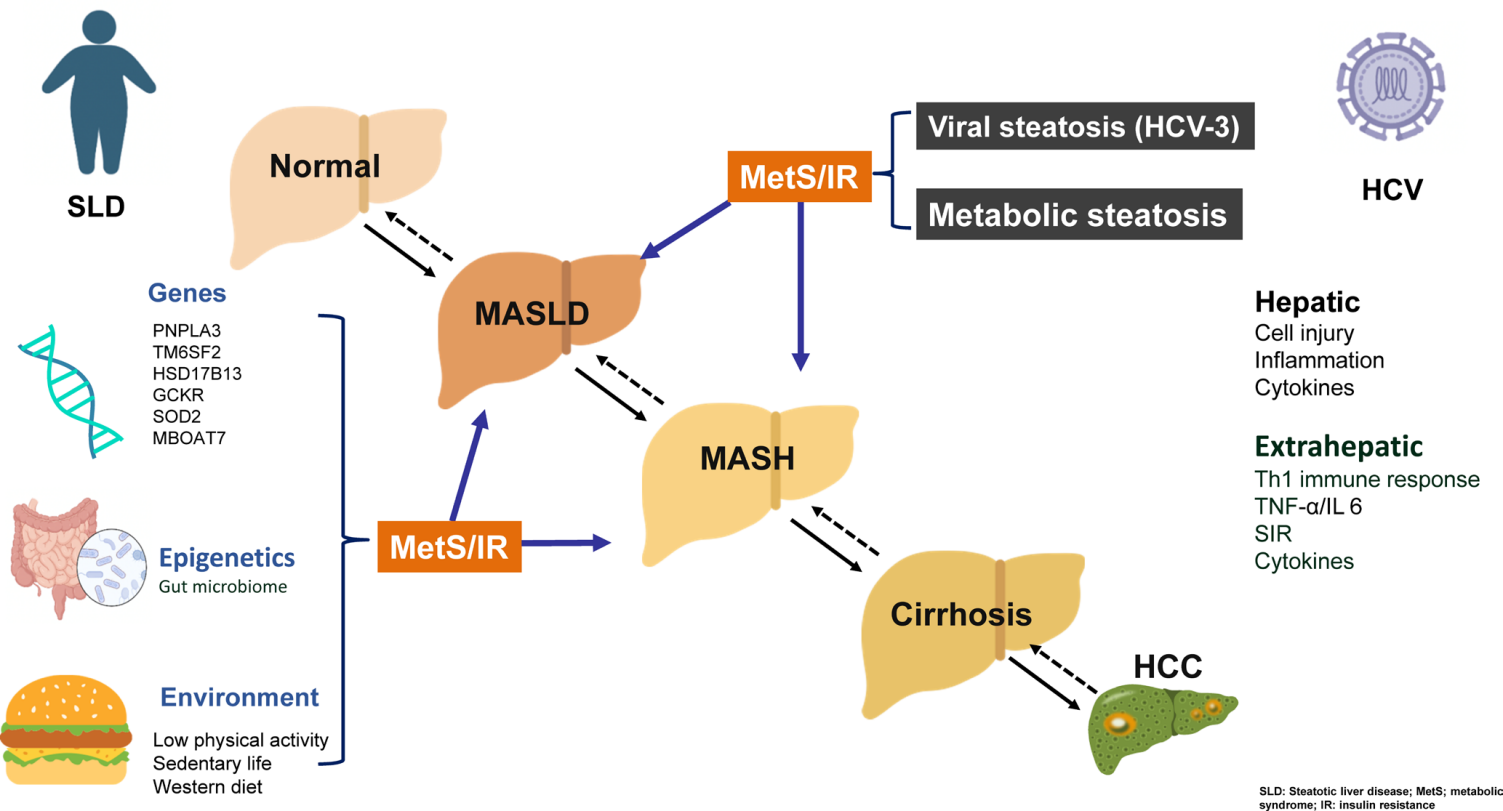
The pathogenic mechanisms leading to the development of liver complications between steatotic liver disease and chronic hepatitis C. Besides liver necroinflammation, HCV triggers immune cascades mainly mediated by Th1 lymphocytes. These lymphocytes increase the activation of TNF‐a and elevation of interleukin‐6 levels. HCV directly leads to steatosis, particularly in those with genotype‐3 infection. Meanwhile, HCV might also induce a systemic inflammatory response and cytokine storms, which are potentially steatotic and fibrogenic, further leading to the development of insulin resistance, which plays a pivotal role in the development of subsequent metabolic abnormalities. Conversely, diabetes, dyslipidemia, obesity, and hypertension are the main features in MASLD, all of which can be modified by genetic, epigenetic, and environmental factors. CHC, chronic hepatitis C; G‐3, genotype‐3; HCC, hepatocellular carcinoma; HCV, hepatitis C virus; IL, interleukin; IR, insulin resistance; MASLD, metabolic dysfunction‐associated steatotic liver disease; MetS, metabolic syndrome; MASH, metabolic dysfunction‐associated steatohepatitis; SIR, systemic inflammatory response; TNF, tumor necrosis factor.

Hepatitis C virus (HCV) infection is the major cause of cirrhosis and hepatocellular carcinoma and has a huge impact on public health globally [1, 2]. Besides liver injury, HCV is lymphotropic. Replication of HCV in diseased extrahepatic tissues might lead to extrahepatic metabolic disorders [3]. Hepatic steatosis is a common manifestation of chronic hepatitis C infection (CHC), most commonly found in patients with genotype‐3 (G‐3) infection, possibly due to direct effects of genotype‐specific viral proteins [4]. In patients infected with other genotypes, hepatic steatosis is mostly due to the interference in host insulin metabolism. Hepatic steatosis correlates with viral load, is mitigated after achieving the sustained virological response (SVR), and might recur in G‐3 patients who relapsed from treatment. Meanwhile, CHC could lead to metabolic alterations such as type 2 diabetes mellitus, cardiovascular diseases (CVDs) and dyslipidemia [5]. The pathogenic mechanisms leading to the development of liver complications between steatotic liver disease (SLD) and CHC are demonstrated in Figure 1. The clinical presentations of the metabolic abnormalities may change after successful HCV eradication and subsequent changes of HCV‐related metabolic alterations, especially SLD with the possession of cardiometabolic risk factors (CMRFSs) after viral eradication, is an interesting issue for review and discussion.

## 2 Prevalence of Hepatic Steatosis in Chronic Hepatitis C

HCV cell entry is engaged by cellular proteins such as low‐density lipoprotein (LDL) receptor, tetraspanin CD81, claudin‐1, occludin, etc. Meanwhile, lipoproteins possess the determining impact on the process of HCV infection by promoting endocytosis of HCV lipoprotein receptors [6], so alteration of blood lipids and the resulting hepatic steatosis are characteristic features of HCV infection. The prevalence of liver steatosis ranges from 35% to 70% in CHC patients, which is significantly higher than in patients with other chronic liver diseases and the general population [7, 8, 9, 10, 11]. The high proportion of hepatic steatosis in CHC patients could be attributed to metabolic disturbance of insulin resistance or direct insult of viral‐specific steatosis of HCV G‐3 infection [4, 12].

It is estimated that the global prevalence of SLD is increasing as a whole [13]; similarly, the prevalence of SLD in CHC has been consistently reported [8, 11]. The universal increasing prevalence is not just due to increasingly sedentary lifestyles and processed diets in recent decades but to the rapid Westernization on a global scale as well. The features regarding the prevalence of SLD in CHC might be different subsequent to different SLD‐associated genetic predispositions across ethnicities [14], the proportion of HCV G‐3 infection, and the definitions of hepatic steatosis.

The most reliable methodology is from histology. A large‐scale study using 1018 biopsy‐proven data in Taiwan where HCV G‐3 accounted for < 5% showed that the prevalence of hepatic steatosis was 47% in this sample. Body mass index (BMI) and patatin‐like phospholipase domain‐containing 3 (PNPLA3) genetic polymorphism interactively determined the presence and steatotic degree [11]. Notably, hepatic steatosis might aggravate liver fibrosis progression in the early disease course and decrease its component later in the natural course due to burnout phenomenon in subjects with SLD [15]. There is a trajectory presentation regarding the proportion of hepatic steatosis from fibrosis stage 0 (F0) to stage 4 (F4). Huang et al. have reported an increased proportion of hepatic steatosis from F0 (20%), F1 (40%) to F2 (63%), but it decreased from F3 (55%) to F4 (41%) [16]. Consequently, liver disease severity should be taken into consideration in the estimation of the hepatic steatosis prevalence in a target population.

## 3 Nomenclature of SLD in CHC

In the era of nonalcoholic fatty liver disease (NAFLD), all other concurrent etiologies of liver disease including HCV infection are excluded. With the emerging terminology of metabolic dysfunction‐associated fatty liver disease (MAFLD) throughout 2020 to 2023, HCV‐associated fatty liver in accordance with the presence of metabolic dysfunction is encompassed in the discussion of SLD. As a result, MAFLD patients tend to have poor liver and nonliver‐related outcomes compared to NAFLD ones, which is largely attributed to distinct metabolic disarrangement and coexisting other liver diseases including CHC [17, 18]. The interaction of HCV infection and MAFLD could be further addressed under the umbrella terminology [19]; for instance, the lean MAFLD subtype has been independently associated with significant liver fibrosis in a viral hepatitis endemic area [20]. MAFLD with diabetes and metabolic dysfunction subtypes could aggravate liver fibrosis in subjects without CHC [21].

Recently, major international societies have postulated the umbrella term of SLD [22]. Metabolic dysfunction‐associated steatotic liver disease (MASLD) is a group of SLD patients carrying at least one item of CMRFs that include (1) BMI ≥ 23 kg/m^2^; (2) fasting plasma glucose ≥ 100 mg/dL, hemoglobin A1c (HbA1c) ≥ 5.7% or type 2 diabetes history with or without treatment; (3) blood pressure ≥ 130/85 mmHg or specific antihypertensive drug treatment; (4) plasma triglycerides ≥ 150 mg/dL or lipid‐lowering treatment; and (5) plasma high‐density lipoprotein cholesterol ≤ 40 mg/dL for males and ≤ 50 mg/dL for females or lipid‐lowering treatment.

Due to IR and chronic inflammatory status, the possession of CMRFs in CHC accounts for a higher risk of cardiocerebrovascular events than the general population [23, 24]. Notably, once CHC patients have SLD, more than 90% of them carry at least one CMRF [9, 10, 25, 26]. Despite the close linkages between HCV infection and SLD as well as cardiovascular risks, the endorsed definition of SLD categorizes HCV‐related steatosis as “miscellaneous SLD.” [22] By enrolling a nationwide CHC cohort, Huang et al. disclosed that SLD patients who carry CMRFs were at greater risk of both CVD and advanced liver fibrosis as one‐third of the CHC patients met the diagnostic criteria of MASLD [25]. The clinical characteristics shared the similarity of NAFLD presentations in the general population; therefore, CHC patients with SLD and CMRFs should be viewed as “HCV‐MASLD” [9].

## 4 Change of SLD Status, CMRFs Before and After HCV Cure

Obesity is the main factor contributing to the development of CMRFS. Significant body weight gain after successful viral eradication has been observed in previous studies due to potential improvement of quality of life and improvement of systemic inflammation. However, discordant results addressing no change or a reduction in body weight have also been depicted in a previous study [27]. Notably, since BMI data reflect normal population distribution, a subtle change would be statistically significant in a study comprising a large sample size [25].

HCV eradication improves IR, mitigates beta‐cell function while further facilitating glycemic control [3, 27]. Concordant study observations revealed improvement of glycemic index and reversal of hypolipidemia after achieving SVR [3, 21, 27, 28, 29]. SVR achieved by DAAs is associated with long‐term improvement of glycometabolic control in diabetic CHC patients, yielding 74.5% patients achieving ≥ 0.5% decrease in HbA1c levels and 33.3% patients having de‐escalation of insulin therapy or switch to oral therapy [30]. Additionally, SVR decreases the incidence of diabetes complications such as acute coronary diseases, ischemic stroke, end‐stage renal disease and retinopathy [31]. Consequently, regional guidelines from Asia‐Pacific regions have listed the benefits of diabetes patients with CHC who have achieved SVR. Such benefits have included at least: 1. Reducing the risks of diabetes complications such as CVD, ischemic stroke, nephropathy, and retinopathy; 2. Reducing the health and economic burden due to HCV infection, diabetes, and their associated comorbidities.; and 3. Improving glycemic and metabolic control [32].

The release of entrapped lipoproteins from the liver after HCV eradication reverses the characteristic pan‐hypolipidemia in CHC. A recent meta‐analysis showed that there was an increase in total cholesterol, low‐density lipoprotein cholesterol (LDL‐C), and high‐density lipoprotein cholesterol but not triglycerides. Notably, there would be a tradeoff effect between the improvement of glycemic control and elevated LDL‐C with respect to the magnitude of cardiocerebral risk reduction after HCV eradication [23, 24, 33].

A series of studies denoted the change of hepatic steatosis before and after HCV eradication. While some studies disclosed there was no change or resolution of hepatic stenosis, others proposed newly developed SLD after HCV eradication [10, 25, 27]. The different results could be attributed to different patient characteristics, inclusion definitions of hepatic steatosis, or varying follow‐up periods after antiviral therapy.

## 5 Dynamic Change of MASLD Status After HCV Cure

The dynamic changes of the MASLD before and after HCV eradication deserve clarification in terms of the changes of CMRFs and SLD status. Liu et al. evaluated 1512 CHC patients with SVR by measuring the controlled attenuation parameter value via vibration‐controlled transient elastography as well as CMRFs before treatment and 12 weeks after the end of treatment, where the prevalence significantly decreased from 45.0% to 36.1% [10]. By contrast, Huang et al. conducted a nationwide study by enrolling 5840 direct‐acting antiviral (DAA)‐treated patients with available sonography‐defined SLD and CMRFs. The prevalence of MASLD did not significantly change 6 months after HCV cure [25]. Further analysis demonstrated that the proportion of patients with MASLD resolution in baseline MASLD patients did not differ between the two studies, but the proportion of MASLD emergence in baseline non‐MASLD subjects significantly increased. The emergence of MASLD was largely attributed to SLD subjects who eventually developed at least one CMRF after SVR. The discordant results between studies might be due to the differences in terms of patient characteristics, disease definitions, and observational periods. Pretreatment metabolic traits could largely determine the evolution of MASLD; to wit, CHC patients with higher BMI, hemoglobin A1C and LDL‐C had a higher risk for developing MASLD, whereas patients with lower pretreatment BMI had higher chances of MASLD resolution [25]. The presence of diabetes might have also contributed to MASLD development in one observational study [10].

## 6 Conclusions

CHC patients have a higher risk of SLD and CMRFs compared to other etiologies of chronic liver diseases. The terminology of MASLD should be appropriately modified in CHC patients with SLD and CMRFs, which are associated with a high risk of liver fibrosis and CVD. Improvement of glycemic index and the reversal of hypolipidemia could be observed after successful HCV eradication; nonetheless, the changes in BMI and MASLD after HCV cure in CHC patients deserve exploration in a long‐term follow‐up manner.

## 7 Expert Opinions

The extremely high efficacy, high safety, short treatment duration, low adverse effects, and easy dosing of DAAs have drastically changed the landscape of HCV treatment in the past one to two decades. It is an excellent story of medical therapeutics in human history. The MASLD changes existing behind the successful story of HCV cure are an important issue in a clinical setting. Of note, the presence of CMRFs that could increase CVD and fibrosis risks in CHC patients with MASLD cannot be stratified as a “miscellaneous SLD” group, and this special population should be viewed as HCV‐MASLD.

Holistically, there was no significant status transition of MASLD after SVR despite a substantial proportion of patients developing or resolving SLD, and this nonsignificance might be attributed at least to one of the following: (1) The potential irreversibility of CMRFS upon its development; (2) The heterogeneity of CMRFS which might lack precise weighting according to CVD risk; or (3) The increase of lipid profile after HCV cure as pan‐hypolipidemia is a characteristic phenomenon of HCV infection, mainly by the altered mechanisms of lipid metabolism via HCV. Consequently, CMRFS surveillance is essential for CHC patients with metabolic alterations after SVR where the impact of the reverse and/or the resume of the lipid levels in a proportion of CHC patients after SVR on the CMRFS changes should be monitored, and although this issue might be beyond the current definition of MASLD and CMRFS, it should be viewed in a long‐term manner in terms of CVD risk and events.

HCV eradication improves IR, mitigates beta‐cell function, and decreases the incidence of diabetes complications. The observation further proves the tight link between an infectious disease and a metabolic disorder. The diabetogenic characteristic of HCV also emphasizes the importance of HCV screening in diabetes patients; meanwhile, HCV treatment in the most timely manner is mandatory in CHC patients with diabetes. Further studies are warranted to address the long‐term hepatic and cardiovascular outcomes for patients who have altered MASLD status in the postviral eradication era.

## Funding

The paper was partly sponsored by Kaohsiung Medical University (KMU‐TB114003, KMU‐TC114A08) and Kaohsiung Medical University Hospital (KMUH‐DK(B)‐114004–1).

## Conflicts of Interest

Chung‐Feng Huang: Speaker for AbbVie, BMS, Bayer, Gilead, Merck, and Roche. Jee‐Fu Huang: Research Grant from Gilead, Bristol‐Myers‐Squibb. Consultant of Roche, Sysmex, Boehringer Ingelheim, and Aligos. Speaker for AbbVie, Gilead, Merck, Sysmex, and Novo Nordisk. Ming‐Lung Yu: Research grant from Abbott, BMS, Merck, and Gilead; Consultant of AbbVie, Abbott, Ascletis, BMS, Merck, Gilead, and Roche; Speaker for AbbVie, Abbott, BMS, Merck, Gilead, and IPSEN. Wan‐Long Chuang: Consultant of Gilead, AbbVie, BMS, PharmaEssentia, and Aligos; Speaker for Gilead, AbbVie, BMS, and PharmaEssentia. The authors have no other relevant affiliations or financial involvement with any organization or entity with a financial interest in or financial conflict with the subject matter or materials discussed in the manuscript. This includes employment, consultancies, honoraria, stock ownership or options, expert testimony, grants or patents received or pending, or royalties.

## Data Availability

The data that support the findings of this study are available on request from the corresponding author. The data are not publicly available due to privacy or ethical restrictions.
